# Dedifferentiated chondrosarcoma: Radiological features, prognostic factors and survival statistics in 23 patients

**DOI:** 10.1371/journal.pone.0173665

**Published:** 2017-03-16

**Authors:** Chenglei Liu, Yan Xi, Mei Li, Qiong Jiao, Huizhen Zhang, Qingcheng Yang, Weiwu Yao

**Affiliations:** 1 Department of Radiology, Shanghai Jiao Tong University Affiliated Sixth People’s Hospital, Shanghai, China; 2 Department of Pathology, Shanghai Jiao Tong University Affiliated Sixth People’s Hospital, Shanghai, China; 3 Department of Orthopedics, Shanghai Jiao Tong University Affiliated Sixth People’s Hospital, Shanghai, China; Universite de Nantes, FRANCE

## Abstract

**Background:**

Dedifferentiated chondrosarcoma is a rare, highly malignant tumor with a poor survival. There are many confusing issues concerning the imaging feature that can facilitate early diagnosis and the factors that might be related to outcomes.

**Methods:**

Twenty-three patients with dedifferentiated chondrosarcoma confirmed by pathology were retrospectively reviewed from 2008 to 2015. The patients’ clinical information, images from radiographs (n = 17), CT (n = 19), and MRI (n = 17), histological features, treatment and prognosis were analyzed.

**Results:**

There were 12 males and 11 females, and the mean age was 50.39 years old. Fourteen cases affected the axial bone (pelvis, spine), and 9 cases involved the appendicular bone. Seven (41.17%), 9 (47.36), and 12 (66.66%) lesions showed a biphasic nature on radiograph, CT and MRI, respectively. Of the lesions, 17.39% (4/23) were accompanied by pathological fractures. Histologically, the cartilage component was considered histological Grade1 in 12 patients and Grade 2 in 11 patients. The dedifferentiated component showed features of osteosarcoma in 8 cases, malignant fibrous histiocytoma in3 cases, myofibroblastic sarcoma in 1 case and spindle cell sarcoma in 11cases. Twenty-two cases were treated with surgical resection, and 17 cases achieved adequate (wide or radical) surgical margin. In 8 cases, surgery was combined with adjuvant chemotherapy. The overall median survival time was nine months; 17.4% of patients survived to five years.

**Conclusion:**

Axial bone location, lung metastasis at diagnosis, inadequate surgical margin, incorrect diagnosis before surgery and pathological fractures was related to poorer outcome. Pre- or postoperative chemotherapy had no definitively effect on improved survival.

## Introduction

Dedifferentiated chondrosarcoma is a rare, highly malignant variant of chondrosarcoma, comprising up to 11% of all chondrosarcomas[1]. This term was first described by Dahlin and Beabout in 1971[2]. It is characterized by a high-grade, non-cartilaginous sarcoma juxtaposed with a low or intermediate chondrosarcoma with an abrupt interface between these two components [3]. The dedifferentiated component can demonstrate various features, such as osteosarcoma, malignant fibrous histiocytoma(MFH), angiosarcoma, fibrosarcoma, rhabdomyosarcoma, leiomyosarcoma, anaplastic spindle cell sarcoma, and giant cell tumor [4]. The age of onset most frequently ranges from 50 to 60 years old with a slight predominance of men, and the most commonly involved sites are the femur, pelvis, and humerus [2, 5]. It exhibits aggressive behavior and generates a high mortality rate compared with conventional chondrosarcoma or non-cartilaginous sarcomatous component alone, with which 5-year survival rates have been less than 20% [6, 7]. It was believed that the improved survival would be attained by prompt, correct pretreatment diagnosis and surgical resection with wide or radical margin[8]. Clinically, the doctor is suspicious of this disease based on careful analysis of radiographic features and the clinical course[9].

Dedifferentiated chondrosarcoma can have a wide range of imaging features. Identifying these imaging features is not only essential to raise suspicious of this lesion, but also to help guide choice of preoperative biopsy site or multifocal sampling at initial diagnosis. Because the dedifferentiated component determines the growth of lesion, metastasis, and prognosis of patients[4], it is particularly important to identify imaging features of dedifferentiation underlying chondroid lesion. Radiographs demonstrating aggressive osseous destruction or cortical infiltration, large unmineralized soft tissue mass, pathological fracture, area with osteoid matrix and rapid progression were suggestive of dedifferentiation[10]. Mercuri et al further described its radiographic features and classified into three types for convenience of description and diagnosis [11]. However, not commonly, radiographs may be no evidence of chondral matrix mineralization and dedifferentiation. Some authors had been reported the MRI feature of dedifferentiated chondrosarcoma, who noted that region of reduced signal intensity compared with hyper intensity chondral component was corresponded to dedifferentiation on T2 weighted spin echo or short T1 inversion recovery sequences based on small series [12–14]. Some arthors also reported that the biphasic nature (i.e., distinctly diverse tumor features juxtaposed with chondroid lesions) may be a special and typical feature for dedifferentiated chondrosarcoma [15–17]. However, the tumor showed a biphasic nature on only one-third of radiographs, one-third of MRI images and one-half of CT scans [16].

Currently, surgical resection with disease-free margins and limb salvage remain a preferred treatment for dedifferentiated chondrosarcoma[18]. Authors reported that the outcome was favorable with a radical resection, and local recurrence was related to inadequate surgical margin [6–8, 18]. However, it is rather difficult to implement wide surgical resection for dedifferentiated chondrosarcoma because of pathological fractures, lesion permeability, skip lesions and frequent secondary lung metastasis[15]. Grimer RJ et al noted that 27% cases fail to have adequate (wide or radical) surgical margin, and patients with axial tumors had an apparent increase in inadequate margins as opposed to patients with appendicular lesions [7]. Usually, the behavior of chondrosarcoma was resistant to available chemotherapy and radiation. The presence of dedifferentiated component raised the possibility of neo-adjuvant or adjuvant chemotherapy and radiation[8]. Radiation is associated with several side effect and long term complication, which may increase the risk of developing undifferentiated sarcoma[19, 20]. So, palliative radiation was usually available for patients with chondrosarcoma that are not resectable and cause complaints, especially mesenchymal chondrosarcoma.

Chemotherapy may be an adjunctive therapeutic strategy for dedifferentiated chondrosarcoma. However, because there have not been many reports about this rare disease, the role and effect of chemotherapy in dedifferentiated chondrosarcoma remain uncertainty [6, 8, 18, 21, 22]. A report from Mitchell et al noted that six patients underwent postoperative chemotherapy, the survival at five year was about 36%, whereas, additional six patients underwent only resection, without chemotherapy, all of them died within one year. The patients who received chemotherapy had a favorable outcome than those who did not [8]. However, Dickey et al noted that patients received adjuvant therapy whose median survival time was 7.5 months, and the five year rate of disease–free survival was 7.1%. There was no significant difference in the five year rate of disease–free survival concerning the use of chemotherapy [22]. Additionally, there was no papers documenting many cases received preoperative neoadjuvant chemotherapy achieved a good histological response. Mitchell et al noted that five patients underwent preoperative cytotoxic chemotherapy, only one achieved significant necrosis (>90%) [8]; Staals et al had no good responder in four patients [6], and Dickey et al reported poor chemotherapy response in 21patients who received preoperative neoadjuvant chemotherapy [22].

Identifying the potential prognostic factors of dedifferentiated chondrosarcoma was very important to patients themselves and doctors due to limited treatment options, which may help doctors to make decisions concerning treatment with future perspective. However, the potential prognostic factors remain unclear until now. Staals EL et al. reported that the factors, such as metastatic disease at diagnosis, MFH dedifferentiation, and a high proportion of dedifferentiated components in lesions, were associated with poor survival rates [6]. However, Grimer RJ et al showed that poor prognostic factors were the presence of a pathological fracture at diagnosis, a pelvic location and increasing age, whereas the histological subtype did not significantly affect outcome [7]. To increase recognition of this rare disease, we reported our experience of diagnosing and treating dedifferentiated chondrosarcoma in 23 patients, and we have attempt to further determine which factors that might be related to the outcomes.

## Materials and methods

Our retrospective study was approved by the hospital institutional review board (Shanghai No. 6 People's Hospital), and the need for written informed consent was waived because the study was a retrospective study using a datum from which the patients’ identification had been removed.

A total of 31 patients with a definitive diagnosis of dedifferentiated chondrosarcoma at our institution between 2008 and 2015 were reviewed. Of these cases, 8 patients were excluded due to lack of detailed clinical records and images. Finally, 23 patients were enrolled in our study. All of the patients were treated and followed up in our Department of Orthopedic Oncology. Clinical and follow-up data were obtained from the medical records and the patients themselves.

Patients underwent standard radiographic examinations on a DR (Siemens, Erlangen, Germany). CT scans were performed on a 16-row CT scanner (Siemens Medical Solutions, Erlangen, Germany) or 64-row CT scanner (Light Speed VCT; GE Healthcare, Waukesha, WI) with both bone and soft tissue windows at 2-mm thicknesses and sagittal and coronal reformatted images. MR imaging was performed on 3.0-T superconducting MR scanner (Koninklijke Philips NV, Amsterdam, the Netherlands).

All of the imaging findings were reviewed by two musculoskeletal radiologists who had 20 years of image diagnostic experience and complete knowledge of this tumor (Weiwu Yao, Mei li). If the diagnostic outcome was different between the two observers, an agreement was reached by consultation. The radiographic studies included the following: patterns of bone destruction; the presence and pattern of periosteal reaction; pathologic fracture; matrix mineralization; the presence and features of soft-tissue masses; pattern of enhancement and signal intensity characteristics on MR imaging; and the presence of biphasic nature. The biphasic nature was defined as demonstrating distinctly diverse tumor features adjacent to chondroid lesions, such as dominant lytic area within a mineralized tumor, a large unmineralized soft tissue mass, and a region with osteoid matrix[16]. The histologic slides were stained with hematoxylin and eosin, and when indicated, phosphotungstic acid hematoxylin (PTAH) and periodic acid-Schiff (PAS) were used. The histological features, including the histologic grade of the cartilaginous component and histologic subtype of the dedifferentiated component, were reviewed by two pathologists with more than 20 years of experience to ensure reliability in classification and grading (Huizhen Zhang, Qiong Jiao). The histological subtype depended on immunohistochemistry.

All of the patients were followed up to death or at least for 12 months. For survival analysis, Kaplan-Meier survival analysis was used. Differences between groups were analyzed using the log-rank test. All of the statistical tests were 2-sided, and P values <0.05 were considered to statistical significance.

## Results

### Clinical information

The detail clinical information is summarized in Table 1.

**Table 1:** Clinical information about cases with dedifferentiated chondrosarcoma.

Case	Sex	Age	Location	Symptoms(duration)	Treatment	Margin of resection	Recurrence	Time to lung metastasis	Follow-up(months)
1	M	69	Lt.prox.fem	Pain6m	EPR+c+r	wide	n	2m	DOD5m
2	F	46	Rt.mid.hum	Mass36m	ERP	wide	n	n	AWD60m
3	F	65	Rt.prox.hum	Pain1m	ERP+c	wide	n	Initial;Liver1m	DOD8m
4	M	73	Lt.dist.femur	Pain4m	ERP+c	wide	11m	12m;multiple	DOD17m
5	F	42	Lt.prox.hum	Pain6m	curettage+EPR	Intralesional+wide	1m	n	AWD19m
6	M	32	Rt.finger	Pa.fr	amputation	radical	n	n	AWD18m
7	F	36	Lt.prox.hum	Pa.fr	EPR	wide	n	2m	DOD8m
8	M	45	Rt.clavicle	Mass36m	EPR	wide	14m	n	AWD18m
9	F	49	Lt.prox.tibia;Lt.prox.fibula	Pain24m;mass10m	amputation	radical	n	n	AWD67m
10	F	36	Rt.pelvis	Pain6m	He+EPR+c	wide	n	n	AWD27m
11	F	42	Rt.pelvis	Pain12m	He+EPR+c	wide	24m	n	DOD32m
12	F	59	Lt.pelvis	Pain4m	He+EPR	wide	n	initial	DOD6m
13	F	40	Rt.pelvis	Pain12m	He+EPR+c	marginal	5m	n	DOD10m
14	M	53	Rt.pelvis	Pain6m	He+amputation	radical	n	Initial;Bone2m	DOD5m
15	M	58	Rt.pelvis	Pain6m;pa.fr	He+h.d	marginal	1m	1m	DOD2m
16	M	50	Lt.pelvis	Pain5m	He+h.d	wide	n	7m	DOD12m
17	M	50	Lt.pelvis	Pain12m	He+amputation	radical	n	initial	DOD5m
18	M	56	Lt.pelvis	Pain1m	He+h.d	marginal	n	2m;multiple	DOD6m
19	F	51	Lt.pelvis	Mass12m;pa.fr	n	n	n	initial	DOD4m
20	M	45	Lt.pelvis	Pain2m	He+EPR	wide	n	initial	DOD9m
21	F	61	Rt.pelvis	Pain5m	He+EPR	wide	n	n	DOD29m
22	M	50	Rt.pelvis	Pain9m	He+EPR	marginal	2m	Initial;Brain4m	DOD7m
23	M	51	T9-T11	Mass5m	EPR+c	marginal	2m	initial	DOD7m

F, female; M, male; Lt, left; Rt, right; prox, proximal; dist, distal; fem, femur; hum, humerus; EPR, limb-salvage surgery with endoprosthetic replacement of resected bone; He, hemipelvectomy; c, chemotherapy; r, radiotherapy; m, months; DOD, died of disease. AWD; alive with disease; pa.fr, pathologic fracture; h.d. hip disarticulation; n, none.

**Fig 1:**
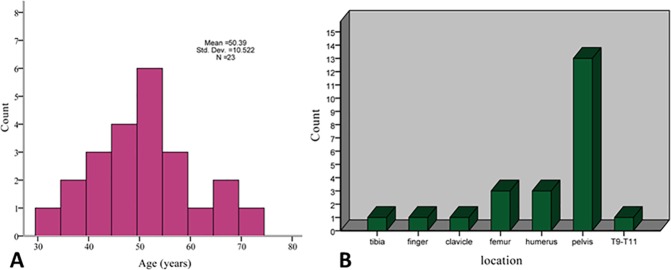
The distribution of age and location in 23 patients with dedifferentiated chondrosarcoma. (A) Age distribution. (B) Location distribution.

The age and location distributions of the patients are shown in Fig 1A and 1B. There were 12 men and 11 women, and the mean age was 50.39 years old (32–73 years). The most frequently involved site was the pelvis (13 cases), followed by the femur (3 cases) and humerus (3 cases). Fourteen cases affected axial bone (pelvis, spine), while 9 cases involved appendicular bone. Twenty-one patients presented a history of pain with duration from 1 month to 12 months, 4 cases reported a history of a mass lasting from 10 months to 36 months, and 4 cases showed pathologic fractures.

### Imaging characteristics

Radiographic imaging data, including radiographs (n = 17), CT scans (n = 19), and MRI studies (n = 17), were obtained for review in 23 patients.

#### Bone destruction, periosteal reaction and pathologic fracture

**Fig 2:**
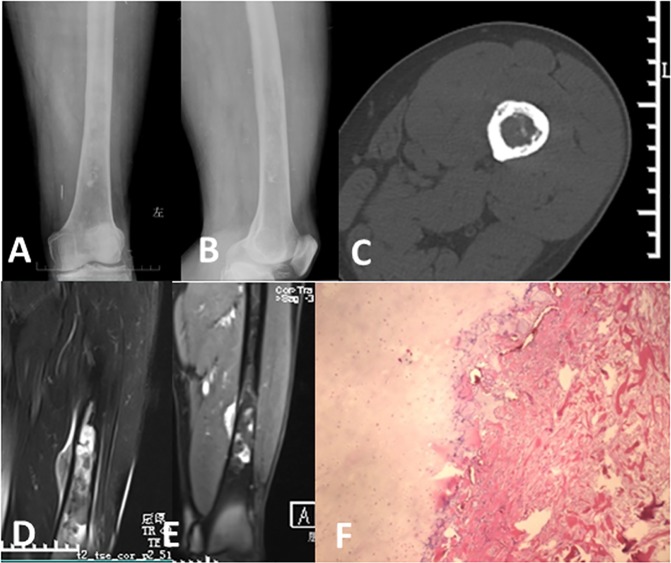
Dedifferentiated chondrosarcoma of the left distal femur in a 73-year-old man (Case 4). (A,B). Anteroposterior and lateral radiographs demonstrated chondral matrix mineralization, showing a low-grade chondral tumor but no signs of cortical destruction. (C). CT showed permeating cortical destruction. (D). Fat-suppressed axial T2-weighted image showed extra-osseous soft tissue with reduced signal intensity, prompting a dedifferentiated component. (E). Enhanced MRI showed punctate, ring or septal enhancement in the intraosseous chondral tumor component and heterogeneous evident enhancement in the extra-osseous dedifferentiated component.(F). Photomicrograph (HE staining X40) showing the biphasic pattern of low grade chondrosarcoma and high grade osteosarcoma. This patient underwent preoperative and postoperative chemotherapy; however, tumor necrosis of the lesion was only approximately 60% on histology. One year later, the patient suffered from lung and multiple organ metastasis and died after five months.

**Fig 3:**
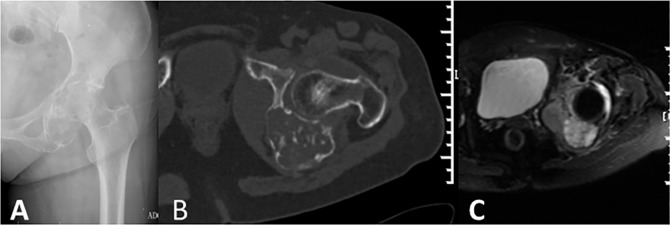
Dedifferentiated chondrosarcoma of the left pelvis in a 59-year-old woman (Case 12). (A). Anteroposterior radiograph demonstrated unilaminar benign periosteal reaction and low-grade chondrosarcoma. (B).CT showed expansive osteolytic bone destruction with typical chondral matrix mineralization and extra-osseous unmineralized soft tissue a mass cluing dedifferentiated component. (C). Fat-suppressed coronal T2-weighted image showed clear demarcation between reduced signal intensity (dedifferentiated component) and chondral signal intensity. The preoperative needle biopsy was accurate based on MRI.

Nine lesions in the appendicular bone showed typical infiltrated cortical destruction on radiographs or CT scans, among which 1 lesion showed permeation though the Haversian system on the CT scan but no aggressive cortical changes on radiographs (Fig 2A–2C), 2 lesions showed moth-eaten cortical destruction, and 6 lesions demonstrated irregular osteolytic bone destruction with soft tissue masses of varying sizes; 13 lesions located in the pelvis and l lesion located in the posterior column of the T9-11 vertebral bones showed expansive osteolytic bone destruction. The host bone maintained the previous bone shape and usually had a well-defined sclerotic margin with or without a wide transition zone. Twenty-one lesions were located in the center of the host lamellar bone, and 2 lesions showed eccentric growth. Two cases revealed a benign periosteal reaction, which manifested as solid, thick, unilaminar new bone disposition on radiographs (Fig 3A), and17.39% lesions (4/23) showed pathologic fractures.

#### Matrix mineralization

**Fig 4:**
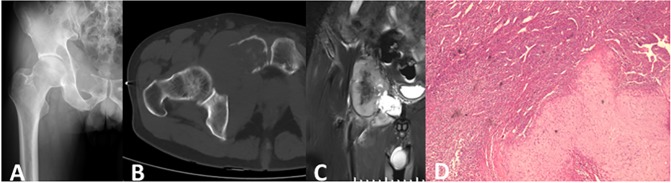
Dedifferentiated chondrosarcoma of the right pelvis in a 58-year-old man (Case 15). (A). Anteroposterior radiograph demonstrated osteolytic bone destruction and a soft tissue mass without chondral or osteoid matrix mineralization. (B). CT showed punctate chondral matrix mineralization in the intraosseous medullary cavity, which might be high-grade chondrosarcoma. (C). Fat-suppressed coronal T2-weighted image showed a clear demarcation between predominant reduced signal intensity (dedifferentiated component) and chondral signal intensity. (D). Pathological section (HE X40) showed feature of the low grade chondrosarcoma and high grade malignant fibrous histiocytoma.

**Fig 5:**
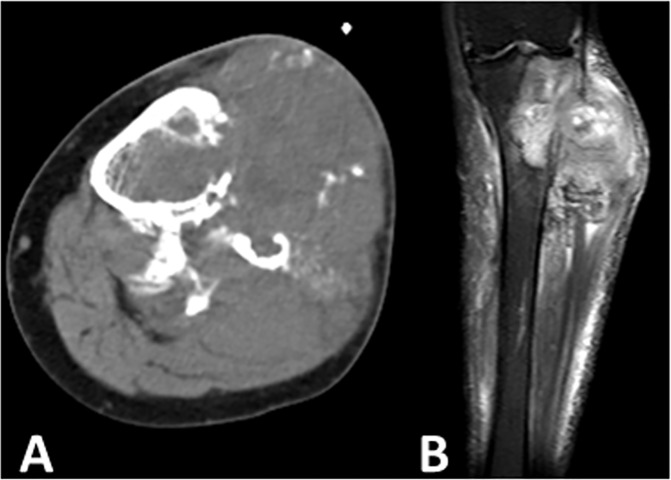
Dedifferentiated chondrosarcoma of the left proximal tibia and fibula in a 49-year-old woman (Case 9). (A).CT showed a flocculent shaped chondral matrix mineralization with marginal irregular shaped ossification and a soft tissue mass (B). Fat-suppressed coronal T2-weighted image showed heterogeneous signal intensity.

Twenty lesions showed chondral matrix mineralization on radiographs or CT scans, among which 2 cases showed no matrix mineralization on radiographs but had intraosseous calcification on CT scans (Fig 4A and 4B); only 3 lesions demonstrated no evidence of matrix mineralization. Sixteen lesions displayed punctate shaped chondral matrix mineralization, 2 lesions displayed ring and arc shapes with extension along the longitudinal axis of diaphysis, 1 lesion showed a flocculent shape with marginal, irregularly shaped ossification (Fig 5A), and 1 lesion was located in the posterior column of the T9-11 vertebral bones and also showed a large soft tissue mass with punctate and patchy calcification. The chondral matrix mineralization of 18 lesions was situated in the marrow cavity, and 2 lesions were located outside of the marrow cavity.

#### Soft tissue mass

An associated soft tissue mass was shown on 58.82% (10/17) of radiographs, 84.21% (16/19) of CT scans, 94.11% (16/17) of MRI images. CT scans and MRI images of 15 cases were available, three soft tissue masses were detected on MRI image but were not shown on CT scans. The soft tissue masses displayed different sizes, the smaller showed nodular protrusion adjoining the permeating bone destruction (Fig 2D), whereas the larger displayed soft tissue masses without matrix mineralization and had clear demarcation with neighboring soft tissue (Fig 4B and 4C).

#### Signal intensity and enhancement characteristics on MRI images

Twelve of 17 cases displayed an area of atypically reduced signal intensity next to feature of low grade chondrosarcoma which showed lobulated, uniformly hyper intensity with ring or arc low signal intensity septal, apart from areas of matrix mineralization, which displayed foci of signal void on T2 weighted sequences (Fig 3C, Fig 4C), the other five cases displayed inhomogeneous reduced signal intensity and without typical feature of chondroid tumor (Fig 5B). Twelve cases underwent enhanced MRI imaging, 8 cases showed punctate, ring or septal enhancement in the intraosseous compartment and heterogeneous evident enhancement in extra-osseous soft tissue (Fig 2E), and 4 cases showed heterogeneous enhancement in intraosseous and extra-osseous soft tissue.

#### Tumor biphasic nature

The tumor biphasic nature is summarized in Table 2. The biphasic nature was observed in 7 (41.17%), 9 (47.36), and 11 (64.70%) lesions on radiograph, CT and MRI, respectively.

**Table 2:** Evidence of tumor biphasic nature.

Tumor Biphasic Nature	Radiographs (n = 17)	CT (n = 19)	MRI (n = 17)
Dominant lytic area next to mineralized tumor	1		
Large unmineralized soft tissue mass next to mineralized tumor	6	8	12
Area with osteoid matrix next to chondriod tumor	0	1	0

### Accuracy of diagnosis before surgery

Twenty-three cases underwent core needle biopsy before surgery, among which 2 cases were diagnosed as spindled cell sarcoma, 4 cases as grade I chondrosarcoma, 5 case as grade II chondrosarcoma and 2 cases as osteosarcoma. Only 10 cases had accurate diagnoses of dedifferentiated chondrosarcoma, which biopsy site included atypical reduced signal intensity On T2WMR or large unmineralized soft tissue mass on CT scan, and area of chondrosarcoma component (Fig 3). We classified our 23 patients into two groups on the basis of whether the true diagnosis was achieved before surgical intervention. Group 1 consisted of patients with accurate diagnosis and proper biopsy site, Group 2 had operations intended to curative of malignancy, but the true diagnosis was not known before surgery. The median survival of 10 patients in group 1 was 29 months, of 13 patients in group 2 was 7 months, There was a significant difference between the two groups(X^2^ = 5.235 P = 0.022). A true diagnosis before surgery was found to have better survival. Additionally, 3 patients with pathological fracture in group 2, whose median survival was only 4 months.

### Histologic findings

Histological slides were obtained from 23 patients. The histological grade of cartilage component and subtypes of dedifferentiation in 23 patients are summarized in Table 3.

**Table 3:** The histological grade of cartilage component and subtypes of dedifferentiation in 23 patients.

Case	Grade	Histologic subtypes
1	1	Spindle cell sarcoma
2	1	Myofibroblastic sarcoma
3	2	osteosarcoma
4	1	osteosarcoma
5	1	Spindle cell sarcoma
6	1	Spindle cell sarcoma
7	2	Spindle cell sarcoma
8	2	osteosarcoma
9	1	osteosarcoma
10	2	osteosarcoma
11	2	osteosarcoma
12	1	osteosarcoma
13	1	Malignant fibrous histiocytoma
14	2	Malignant fibrous histiocytoma
15	2	Malignant fibrous histiocytoma
16	1	Spindle cell sarcoma
17	2	Spindle cell sarcoma
18	2	Spindle cell sarcoma
19	1	Spindle cell sarcoma
20	1	Spindle cell sarcoma
21	2	Spindle cell sarcoma
22	1	osteosarcoma
23	2	Spindle cell sarcoma

Grossly, the cartilage component usually located in intramedullary and showed gray-white, gelatinous, gritty appearance, whereas the dedifferentiated area displayed fleshy, fish meat like appearance, usually, but not always, extra osseous. Dedifferentiated components vary greatly, which may be a very small area or a large area of tumor. Rarely, the tumor only showed the gross feature of chondrosarcoma, the dedifferentiated component could be seen histologically.

Histologically, under the microscope, with a biphasic nature, a high-grade non-cartilaginous sarcoma juxtaposed with a low or intermediate chondrosarcoma with an abrupt interface between these two components was observed in all of the patients. Of these, the cartilage component was considered Grade1 in 12 patients, and it showed feature of Grade 2 in 11 patients, whose median survival was 10 months, 8months respectively. No significant difference in survival time had been found between Grade1 and Grade2 (X^2^ = 0.682, P = 0.409). The dedifferentiated component showed features of osteosarcoma in 8 cases (Fig 2F), malignant fibrous histiocytoma in 3 cases (Fig 4D), myofibroblastic sarcoma in 1 case and spindle cell sarcoma in 11 case. Patients with myofibroblastic sarcoma had been disease-free about 60 months until now, patient with malignant fibrous histiocytoma may be a shorter survival time (median 5 months) than patients with osteosarcoma(median 17 months)and patients with spindle cell sarcoma (median 8 months). However, there was no significant statistics difference between groups(X^2^ = 5.650, P = 0.059).

### Treatment and follow-up

#### Surgery

Twenty-two cases were treated with surgical resection, while only 1 case refused surgery due to initial lung metastasis and poor general condition. Four cases suffered amputation in attempt to obtain a radical margin. In appendicular bone, 6 cases underwent tumor segmental resection with wide margin and prosthetic replacement, 1 lesion had intraosseous curettage and subsequently local recurrence after one month treated by limb sparing resection with wide margin. In the axial bone, 11 cases underwent hemipelvectomy and limb sparing, among which 8 cases received prosthesis implantation, and 3 cases underwent hip exclusion. Of these, wide margin of resection were achieved in 7 cases, marginal margin in 5 cases.

**Fig 6:**
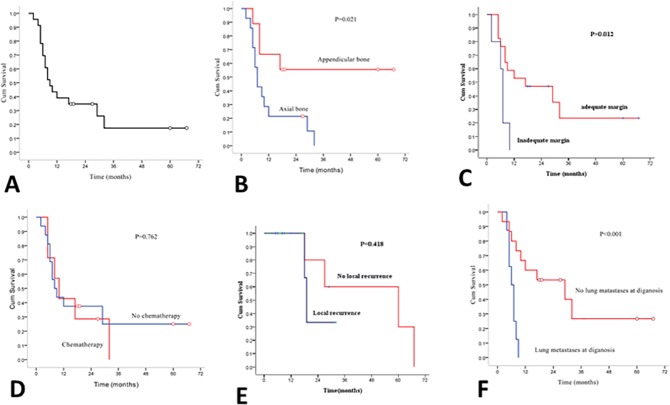
Kaplan-Meier survival curve for 23 cases. (A) The overall median survival time was nine months. (B). Overall survival time for cases in the appendicular bone compared with those in the axial skeleton; the difference is significant. (C). Overall survival time for cases with wide or radical surgical margin compared with inadequate margin; the difference is significant. (D). Overall survival time for cases pre- or post-chemotherapy compared to those without; the difference is not significant. (E). Overall survival time for cases with local recurrence compared to those without; the difference is not significant. (F). The survival curve for cases with lung metastasis at initial diagnosis compared to those without; the difference is significant.

The average duration of follow-up was 16.57 months, the overall median survival time was nine months; 39.1% cases survived to 1 year and only 17.4% patients to five years (Fig 6A). In appendicular bone, the mean survival time was 41.44 months, whereas, in axial bone, it was 11.75 months. There was a significant difference between the two groups (X^2^ = 5.340, P<0.05) (Fig 6B). A total 17 cases achieved wide or wide surgical margin whose median survival was 17 months, whereas 5 cases with marginal margin whose median survival was 7 month. Significant statistic difference had been found between groups (X^2^ = 6.333, P = 0.012) (Fig 6C).

#### Chemotherapy and radiotherapy

Eight cases of our patients had adjuvant chemotherapy or chemotherapy combined with radiotherapy in addition to surgery. Of these, 5 cases received postoperative chemotherapy; 1 case was treated by postoperative adjuvant chemotherapy and palliative radiation (20Gy), 2 cases underwent preoperative neoadjuvant chemotherapy, whereas, the tumor necrosis was less than 60% on histology. There was a wide range of chemotherapy regimens and different courses. The most common drug regimen was cisplatin + epirubicin + iphosphamid. The median survival rate of patients who received postoperative chemotherapy was 10 months and those who did not undergo chemotherapy was 8 months, there was no significant difference in survival time (X^2^ = 0.33 P>0.05) (Fig 6D).

#### Local recurrence

The incidence of local recurrence was closely associated with the surgical margin. Eight of our cases who underwent local tumor resection had a local recurrence. Of these, 5 lesions were located in axial bone, 3 lesions in appendicular bone. Four patients who suffered from recurrence had a marginal margin, 3 patients had wide margin and 1 patient had intralesional margin. Of three patients who had local recurrence with wide margin, the dedifferentiated component was osteosarcoma. The other patients who had a wide or radical surgical margin had no local recurrence. We compared the median survival of patients who suffered from recurrence with those who did not; the median survival time was 60 months and 19 months, respectively. There was no significant difference between two groups (X^2^ = 0.655, P = 0.418) (Fig 6E).

#### Metastasis

Eight cases initially showed lung metastasis, with 6 cases during the treatment course. Multiple organ metastases were observed in 5 cases except for lung metastasis. Patients with metastasis at diagnosis had a shorter median survival time (6 months) than patients without metastasis at diagnosis (29 months). There was a significant difference between the two groups (X^2^ = 11.329, P<0.001) (Fig 6F). The presence of metastasis at the initial diagnosis had a serious effect on the outcomes of patients. Although we employed aggressive surgical resection, there was no evidence of improved survival prognosis.

## Discussion

Our study showed that axial bone location, lung metastasis at diagnosis, inadequate surgical margin, incorrect diagnosis before surgery and pathological fracture was related to a poorer outcome. Histological subtype of dedifferentiation may have effect on survival, whereas the grade of cartilaginous component and local recurrence was not associated with survival. Pre- or postoperative chemotherapy had no definitively effect on improved survival. Any chondroid lesion showing aggressive bone destruction associated with a non-mineralized soft tissue mass or with reduced signal intensity on T2 weighted sequences should be suspected of being dedifferentiated chondrosarcoma for early diagnosis.

In our study, we found that the survival time for axial bone lesions was shorter than for appendicular lesions. This may be associated with inadequate surgical margin. The surgical resection of axial bone lesions is complex and technically demanding [23]. Usually, the size of most axial bone lesions is larger than the appendicular counterpart, and surgical options are restricted due to the tumor being adjacent to vital structures as well as the risk of compromising axial stability [23], which lead to increase rate of local recurrence. In our study, 41.67% (5/12) cases had not achieved wide or radical surgical margin and 41.67%(5/12)cases suffered from local recurrence in axial bone. Whereas, in the appendicular bone, all patients achieved radical or wide margin, and only 22.22% (2/9) cases suffered from local recurrence. This might partially explain lower survival at this location. Our results also confirmed inadequate surgical margin lead a poorer outcome, which was consistent with that of Grimer RJ reported [7]. However, there were numbers of evidence showing local control by surgery may not achieve a better outcome [7, 22, 24]. This may seem to not only emphasize local control, but also control metastases. So, the definitive reason should be further investigated.

The result of our study also showed that lung metastasis at diagnosis was a poor prognostic factor. Malchenko S reported that 90% patients with dedifferentiated chondrosarcoma develop lung metastases within a few months of diagnosis [25]. So, CT scanning of the lungs should be considered an indispensable diagnostic procedure. Currently, the reason of high frequency of dedifferentiated chondrosarcoma metastases remains unclear. It may be involved stem–like cell in the process of tumor’s metastatic dissemination[26]. Chemotherapy may be an option for control metastases. However, there was no convincing evidence of the benefit of chemotherapy. Our result showed pre- or postoperative chemotherapy was not associated with the survival rate. Tumor response was poor to neoadjuvant chemotherapy and could not improve all survival rates, which finding was similar to previous reports [6, 7].

It is well known that the histological grade is an important predictive factor for chondrosarcoma, with low grade chondrosarcoma showing better outcome than high grade tumors [27]. In our study, we compared survival of cartilage component (Grade1) with of Grade 2; No significant differences between the two groups had been found. This result was different from conventional chondrosarcoma, which would indicate that the dedifferentiated component may determine the prognosis of patients. In our series, one patients with myofibroblastic sarcoma had been disease-free about 60 months until now, patient with malignant fibrous histiocytoma may be a shorter survival time (median 5 months) than patients with osteosarcoma(median 17 months)and patients with spindle cell sarcoma (median 8 months). This result suggested possible of histological subtype has effect on survival. Although no significant statistics difference between groups had been found, this may be related to limited number of patients.

In our study, we classified 23 patients into two groups based on whether the true diagnosis was achieved before surgical treatment, the results showed a true diagnosis before surgery has positive effect on survival. The biphasic nature has been said to special and typical features for dedifferentiated chondrosarcoma, [15, 28]. In our study, this feature was shown in 41.17%, 47.36%, and 66.66% of lesions on radiography, CT and MRI, respectively, which was consistent with previous reports[29]. CT has a slight advantage over radiography in detecting intraosseous matrix mineralization and bone involvement of the pelvis or other axial bone. Uncommonly, conventional radiography and CT can fail to show evidence of chondral matrix mineralization and dedifferentiation [28, 30]. In these cases, MRI can be helpful in diagnosis. The chondral component showed lobulated increased signal intensity on T2 due to richness in water; after i.v. injection of contrast material, low-grade chondrosarcoma showed a ring or arc-shaped septal enhancement [31], whereas the dedifferentiated component showed an area of atypically reduced signal intensity, compared to low-grade chondral tissue on T2 weighed sequences with heterogeneous enhancement [13]. In patients with pathological fractures, the imaging findings might be not atypical, which may increase potential challenge for accurate diagnosis. In our series, patients with pathological fracture before true diagnosis had only 4 months lifetime. Considering that dedifferentiated chondrosarcoma had a relatively high rate of pathological fractures, ranging from 13 to 44.4%[4]. In our study, the rate was 17.39% (4/23). Therefore, in all patients with dedifferentiated chondrosarcoma should be counseled about the high risk of pathologic fracture.

It is vital to recognize dedifferentiation before surgery in attempt to plan safe margins of resection and to make best use of adjuvant therapy. Our experience indicated the importance of clinical, radiological, and pathological correction in the diagnosis of suspected dedifferentiated chondrosarcoma. When historical data showed a rapid progression of the disease in a short period of time and radiography showed aggressive bone destruction associated with non-mineralized soft tissue masses or reduced signal intensity on T2 weighted sequence underlying chondroid lesions, the presence of a dedifferentiated component is suggested. Additionally, recognition of the dedifferentiated region was important to preoperative biopsy. It is critical to make sure biopsies should be representative of entire tumor. An inaccurate diagnosis was caused by biopsy depending on non-cartilaginous component alone, without examining image feature for the presence of chondriod lesion, or biopsy relying on cartilaginous component alone, without considering dedifferentiation. For these reason, it is essential to thorough evaluation imaging features and clinical course when interpreting tissue specimens.

There were several limitations of our study. First, there were a small of number cases and limited statistical analysis, which may be result in biased conclusions. Second, not all of patients simultaneously underwent radiography, CT and MR, so we could not directly compare imaging features among multiple modalities. Third, due to the relatively long time span of the retrospective series, we were unable to measure the percentage of dedifferentiated component and were unable to analyze the relationships of the survival rate. Fourth, given there was no difference in survival and a small number of cases, we did not split the lesions into whether they were central or peripheral. Despite these limitations, we believe that our results could provide some help for the more accurate recognition of dedifferentiated chondrosarcoma.

## Conclusion

In summary, we confirmed that dedifferentiated chondrosarcoma remains a fatal disease with dismal survival until now. Axial bone location, lung metastasis at diagnosis, inadequate surgical margin or incorrect diagnosis before surgery and pathological fracture lead a poorer outcome. Histological subtype of dedifferentiation may have effect on survival, whereas the grade of cartilaginous component and local recurrence was not associated with survival. Pre-or postoperative chemotherapy had no definitively effect on improved survival.

CT and MRI are recommended to be the preferred imaging modalities for detecting the features of dedifferentiated chondrosarcoma, especially in the pelvis and another axial bone. MRI may be more value of confirming suspicion and guiding pre–operative biopsy.

We emphasize that importance of clinical, radiological, and pathological correction in the diagnosis of suspected dedifferentiated chondrosarcoma. It is essential to evaluate imaging features and clinical course thoroughly when interpreting tissue specimens.

## Supporting information

S1 Checklist STROBE_checklist_v4_combined_PlosMedicine. (DOCX)

S1 Dataset Minimal data set. (XLSX)
